# Genomic analysis of microRNA time-course expression in liver of mice treated with genotoxic carcinogen *N*-ethyl-*N*-nitrosourea

**DOI:** 10.1186/1471-2164-11-609

**Published:** 2010-10-28

**Authors:** Zhiguang Li, William S Branham, Stacey L Dial, Yexun Wang, Lei Guo, Leming Shi, Tao Chen

**Affiliations:** 1 Division of Genetic and Molecular Toxicology, National Center for Toxicological Research, FDA, Jefferson, AR 72079, USA; 2 Division of Systems Biology, National Center for Toxicological Research, FDA, Jefferson, AR 72079, USA; 3 SABiosciences Corporation, Frederick, MD 21703, USA; 4 Division of Biochemical Toxicology, National Center for Toxicological Research, FDA, Jefferson, AR 72079, USA

## Abstract

**Background:**

Dysregulated expression of microRNAs (miRNAs) has been previously observed in human cancer tissues and shown promise in defining tumor status. However, there is little information as to if or when expression changes of miRNAs occur in normal tissues after carcinogen exposure.

**Results:**

To explore the possible time-course changes of miRNA expression induced by a carcinogen, we treated mice with one dose of 120 mg/kg *N*-ethyl-*N*-nitrosourea (ENU), a model genotoxic carcinogen, and vehicle control. The miRNA expression profiles were assessed in the mouse livers in a time-course design. miRNAs were isolated from the livers at days 1, 3, 7, 15, 30 and 120 after the treatment and their expression was determined using a miRNA PCR Array. Principal component analysis of the miRNA expression profiles showed that miRNA expression at post-treatment days (PTDs) 7 and 15 were different from those at the other time points and the control. The number of differentially expressed miRNAs (DEMs) changed over time (3, 5, 14, 32, 5 and 5 at PTDs 1, 3, 7, 15, 30 and 120, respectively). The magnitude of the expression change varied with time with the highest changes at PTDs 7 or 15 for most of the DEMs. In silico functional analysis of the DEMs at PTDs 7 and 15 indicated that the major functions of these ENU-induced DEMs were associated with DNA damage, DNA repair, apoptosis and other processes related to carcinogenesis.

**Conclusion:**

Our results showed that many miRNAs changed their expression to respond the exposure of the genotoxic carcinogen ENU and the number and magnitude of the changes were highest at PTDs 7 to 15. Thus, one to two weeks after the exposure is the best time for miRNA expression sampling.

## Background

MicroRNA (miRNA) is a class of small nucleic acids that negatively regulate gene expression [1]. They are single-stranded RNA molecules that are not translated into proteins. Each miRNA molecule is partially complementary to one or more mRNA transcripts, and functions to down-regulate gene expression by inhibiting protein translation or destabilizing target transcripts [2]. The expression of miRNAs is regulated developmentally and spatially, and is involved in differentiation and proliferation of cells [3]. Therefore, miRNA molecules can modulate a wide array of growth and differentiation processes in cancer [4]. A number of studies have demonstrated that miRNA expression is commonly dysregulated in human cancer; and that miRNAs are extensively involved in carcinogenesis and act as either dominant or recessive cancer genes [5]. miRNA expression profiling has shown promise in defining tumor malignancy status and is surprisingly informative when used to identify tumor types, differentiation states and developmental lineages [6].

The available information on miRNA function suggests that miRNA expression profiles might also have predictive value for assessing chemical carcinogenicity. It has been reported that expression of some miRNAs is associated with tumor initiation [7]. Thus, specific miRNAs could represent attractive molecules as informative biomarkers of exposure to carcinogens. Studies on the relationship between miRNAs and carcinogen exposure have been previously reported [8] and miRNA expression has been shown to be dysregulated by many carcinogenic agents like 4-(methylnitrosamino)-1-(3-pyridyl)-1-butanone (NNK) [9], 7,12-dimethylbenz[a]anthrance [10], 2-acetylaminofluorene (2-AAF) [11] and radiation [12]. The results of these studies indicate that miRNAs are involved in early stages of carcinogenesis and suggest that miRNAs could be a useful tool for detecting carcinogen exposure. Expression data from these studies, however, were generated primarily from chronic or subchronic carcinogen exposures in which the animals were treated with multiple doses of carcinogens over a long period such as 20 or 24 weeks [9,11]. This kind of studies are able to reveal cumulative effects of chemical treatments on miRNA expression. However, they are unable to provide time course miRNA expression data caused by a single dose of chemical treatment.

Time-course data is an important component of toxicological studies. It reveals a toxicological response as a transient, continuous, or delayed response. Since the regulation of miRNA expression is a dynamic process, a temporal design provides information regarding an appropriate sampling time for miRNA expression change after carcinogen treatment. In this study, *N*-ethyl-*N*-nitrosourea (ENU), a model genotoxic carcinogen, is used for the carcinogen treatment. We treated mice with one dose of ENU and measured the expression level of miRNAs in the liver of the treated and control mice at several posttreatment times. This treatment and sampling design allowed us to analyze the time-course changes of miRNA expression in tissues exposed to the carcinogen. We have previously published the mutant frequency data from mice treated in this study [13]

ENU is mutagenic in a wide variety of mutagenicity test systems and carcinogenic in various organs of mammals [14]. It induces hepatocellular carcinomas in mouse liver that receives a single dose of ENU [15]. Thus, ENU is a suitable genotoxic carcinogen for studying the time course expression of miRNAs. Usually, biological effects of a carcinogen are affected by various factors, including chemical absorption, distribution, metabolism, and elimination. ENU, however, directly alkylates nucleotides without metabolic activation [14]. Thus, choosing ENU as a model carcinogen avoids the effects of these unrelated processes to a large extent due to ENU's direct activity. Most importantly, abundant information on the time-course effects of ENU toxicity and carcinogenicity has been accumulated including data regarding DNA adducts [16], gene mutations [13], gene expression [17] and tumor formation [18]. This information is valuable to understand alteration of miRNA expression induced by ENU exposure.

## Results

### Principal component analysis

**Figure 1:**
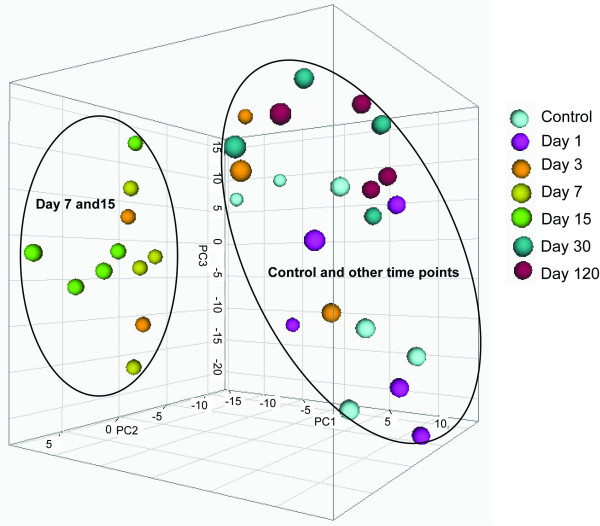
**Principal component analysis of liver samples collected at different times after ENU treatment**. The samples were analyzed according to the expressions of 376 mouse miRNAs using auto-scale method.

To globally view the temporal changes of miRNA expression induced by a single ENU treatment at different posttreatment time points, principal component analysis was performed to classify all of the 35 animal samples (5 samples each group for post-treatment days [PTDs] 1, 3, 7 and 15 after ENU treatment; 4 samples each group for PTDs 30 and 120; and 7 samples for the control group). The normalized Ct values of 376 miRNAs (Additional File 1) were used for this analysis and the results for the first 3 principal components are shown in Figure 1. The samples can be roughly divided into 2 groups. Samples at PTDs 7 and 15 are grouped together while samples from the other groups including the control group and treatment groups at PTDs 1, 3, 30 and 120 are clustered together. The results from the principal component analysis suggest that the miRNA expression was globally altered by ENU treatment at PTDs 7 and 15, but not at PTDs 1, 3, 30 and 120 although two samples at PTD 3 also fall into the PTDs 7 and 15 group, which might reflect that some animals at PTD 3 had miRNA expression patterns similar to those from samples at PTDs 7 and 15.

### Time course analysis of miRNA expression

**Figure 2:**
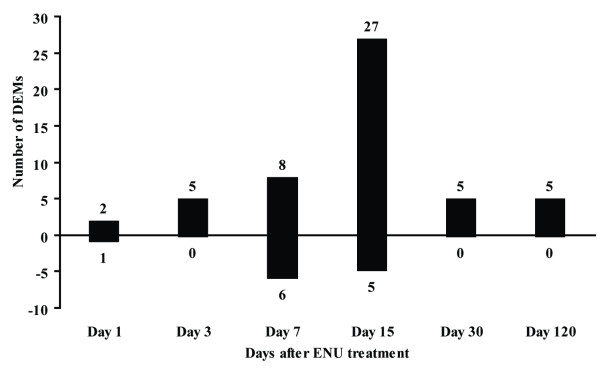
**Number of the differentially expressed miRNAs (DEMs) in livers of mice treated with ENU at different post-treatment days**. The bars above and below x axis represent up- and down- regulated DEMs, respectively. The numbers at the top of each bar denote the amount of DEMs represented by the bar.

There were 43 miRNAs whose expression was dysregulated by ENU treatment at least at one posttreatment time. The names of those differentially expressed miRNAs (DEMs), their fold changes against the controls and the posttreatment time points when their expression was altered, as well as their possible functions reported in the literature are listed in Table 1 (see Additional File 2 for the fold changes and *p *values of all the miRNAs in the PCR array). The amount of the DEMs was changed with time after ENU treatment (Figure 2). Only 3 and 5 DEMs were found at PTDs 1 and 3, respectively. This amount increased to 14 and 32 at PTDs 7 and 15, and then decreased to 5 each at PTDs 30 and 120. Most of the DEMs (4 out of 6) at PTDs 1 and 3 were also observed at PTDs 7 and 15. Two miRNAs, mmu-miR-34a and mmu-miR-34b-5p, were up-regulated at all posttreatment time points except day 120. Most of the miRNAs that were differentially expressed at both PTDs 7 and 15 had the same change directions and similar change magnitudes at the two time points, indicating miRNA alteration during this period was relatively stable (Table 1). However, DEMs at PTD 120 were different from those found in previous posttreatment time points except for mmu-miR-453 that was also dysregulated at PTDs 7 and day 15 but with an opposite change direction (Table 1).

**Table 1:** MicroRNAs whose expressions were significantly changed by ENU in at least one post-treatment time point.

MicroRNA	Day 1	Day 3	Day 7	Day 15	Day 30	Day 120	Function
let-7b				+			Ectopic expression of let-7b reduced HMGA2 expression and cell proliferation in a lung cancer cell line [33].
miR-106b				+			miR-106b was up-regulated in several human tumors compared with adjacent normal tissues and formed a negative-feedback loop with cell cycle regulator E2F1 [43].
miR-130a				+			Expression of miR-130a was significantly up-regulated in primary glioblastomas compared with normal peripheral brain tissue [44].
miR-130b				+			miR-130b showed increased expression in patients with primary WHO grade II gliomas that spontaneously progressed to WHO grade IV secondary glioblastomas [45]. miR-130b was also up-regulated in human T-cell leukemia virus 1 (HTLV-1)-mediated cellular transformation [46].
miR-135b					+ +	+ +	miR-135b expressed was increased in patients with post-surgery elevation of prostate-specific antigen (chemical relapse), as compared with patients with non-relapse disease [47].
miR-138				+			miR-138 suppresses invasion and promotes apoptosis in head and neck squamous cell carcinoma cell lines [34].
miR-144				+			Introduction of miR-144 affected caspase activation in TRAIL-induced apoptosis pathway [48].
miR-150	-						Control of B cell differentiation by targeting the transcription factor c-*Myb *[49].
miR-219				+			miR-219 displayed dysregulated expression in human tongue carcinomas [50].
miR-222			+	+			miR-222 was up-regulated in atypical teratoid-rhabdoid tumors [51].
miR-301a				+			miR-301a expression was significantly differentiated in smoker versus non-smoker [52].
miR-302c*			- - -	- - -			ND
miR-32				+			Over-expression of miR-32 was associated with poor outcome of human kidney cancer [53].
miR-335-5p			+	+			miR-335 was highly expressed in pediatric acute myeloid leukemia [54].
miR-337-5p				+			ND
miR-339-5p		+					ND
miR-34a	+	+ +	+	+ + +	+		Regulation of *p53*-mediated apoptosis [32].
miR-34b-5p	+	+ +	+ + +	+ + +	+ +		Induction of cell cycle arrest by joining *p53 *network [55].
miR-34c		+	+ +	+ +	+		Induction of cell cycle arrest by joining *p53 *network [35].
miR-369-5p			- - -	- - -			miR-369-5p was up-regulated in mesenchymal stem cells propagation [56].
miR-411			-				ND
miR-423-5p		+		+			miR-423-5p was involved in muscle development and growth and showed greatest in the neonate development stage [57].
miR-434-5p			-				ND
miR-451				+			miR-451 expression was up-regulated in multidrug resistant cancer cell lines [58].
miR-453			-	-		+	A variant affecting miR-453's putative target site in estrogen receptor (ESR) 1 is associated with breast cancer risk in premenopausal women [59].
miR-466d-5p				+ +			ND
miR-484				+			miR-484 was involved in adrenal tumorigenesis [60].
miR-487b				+			ND
miR-590-3p			+ + +				miR-590-3p and other miRNAs were suggested to mediate control of autoimmune gene expression [61].
miR-590-5p					+		miR-590 was involved in regulating the expression of transforming growth factor TGF-beta1 and its receptor TGF-betaRII [62].
miR-672			+ +	+			ND
miR-677			+	+			ND
miR-700				+			ND
miR-707				+			ND
miR-762				+			miR-762 was up-regulated in tumor tissue induced by DMBA [10].
miR-871				-			ND
miR-875-3p			- - -				ND
miR-877				+			ND
miR-883a-5p				-			ND
miR-93				+			miR-93 was over-expressed in human T-cell leukemia virus 1- transformed human T-cell lines and primary peripheral blood mononuclear cells from adult T-cell leukemia patients [46].
miR-681						+	ND
miR-205						+	miR-205 expression was down-regulated in breast cancer, but up-regulated in other types of cancer including lung cancer, bladder cancer and ovarian cancer [63].
miR-142-3p						+	miR-142-3p was over-expressed in childhood B-cell precursor acute lymphoblastic leukemia [64].

Note: miRNAs with *p *value < 0.01 and fold change >2.0 were considered significantly changed. The symbols +, ++, and +++; or -, - -, and - - - indicate that the miRNA was up- or down-regulated at a magnitude of 2-4, 4-6, and >6 fold, respectively. No symbol is given if the miRNA was not significantly changed. ND means not determined.

### Confirmation of the temporal expression changes of three miR-34 family miRNAs and one miR-762 family miRNA by individual TaqMan assays

**Figure 3:**
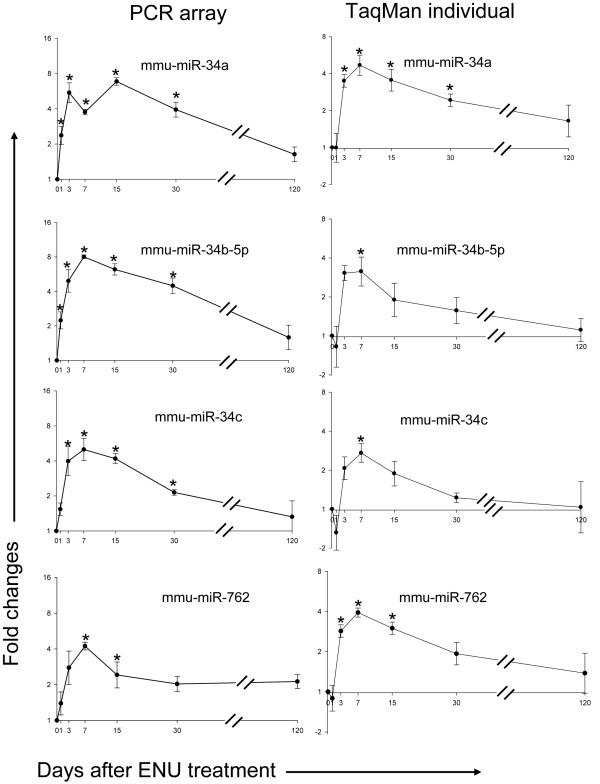
**The temporal expression changes of three miR-34 family members and one miR-762 family member as determined by PCR arrays and individual TaqMan assays**. The data for each time point are the mean of 4 or 5 samples with its standard error. The asterisk indicates there is a significant difference between the treatment and control at that time point (*p *< 0.01).

The miR-34 family is worth special attention because two of its members, mmu-miR-34a and mmu-miR-34b-5p, were significantly up-regulated one day after ENU exposure and maintained increased expression at the 5 subsequent time points up to PTD 30, while another family member, mmu-miR-34c, displayed significant over-expression at multiple time points from PTD 3 to 30. To serve as a confirmation of the data from RT^2^-mouse miRNA PCR Array, a different real-time PCR platform, TaqMan miRNA assay, was used to measure the time-course changes of miR-34 family members induced by the ENU treatment using the same RNA samples. For TaqMan miRNA assay, all of the samples were reversely transcribed simultaneously and the PCRs for the different samples were run in the same plates. This procedure can greatly reduce the inter-plate variability that could be produced when PCR arrays are used. A comparison of miR-34 family miRNA expression measured by the two different platforms is shown in Figure 3. The results from the two real-time PCR assay platforms are very consistent and show similar temporal kinetics of miRNA expression for miR-34 family miRNAs, rising from day 1 or 3, reaching peaks at day 15, and decreasing until the end of observation, day 120. Another miRNA, mmu-miR-762 that is not similar with miR-34 family miRNAs in sequence, were also examined to confirm the array data. The consistency between two platforms was also observed (Figure 3).

### Hierarchical clustering analysis of the DEMs at different posttreatment times

**Figure 4:**
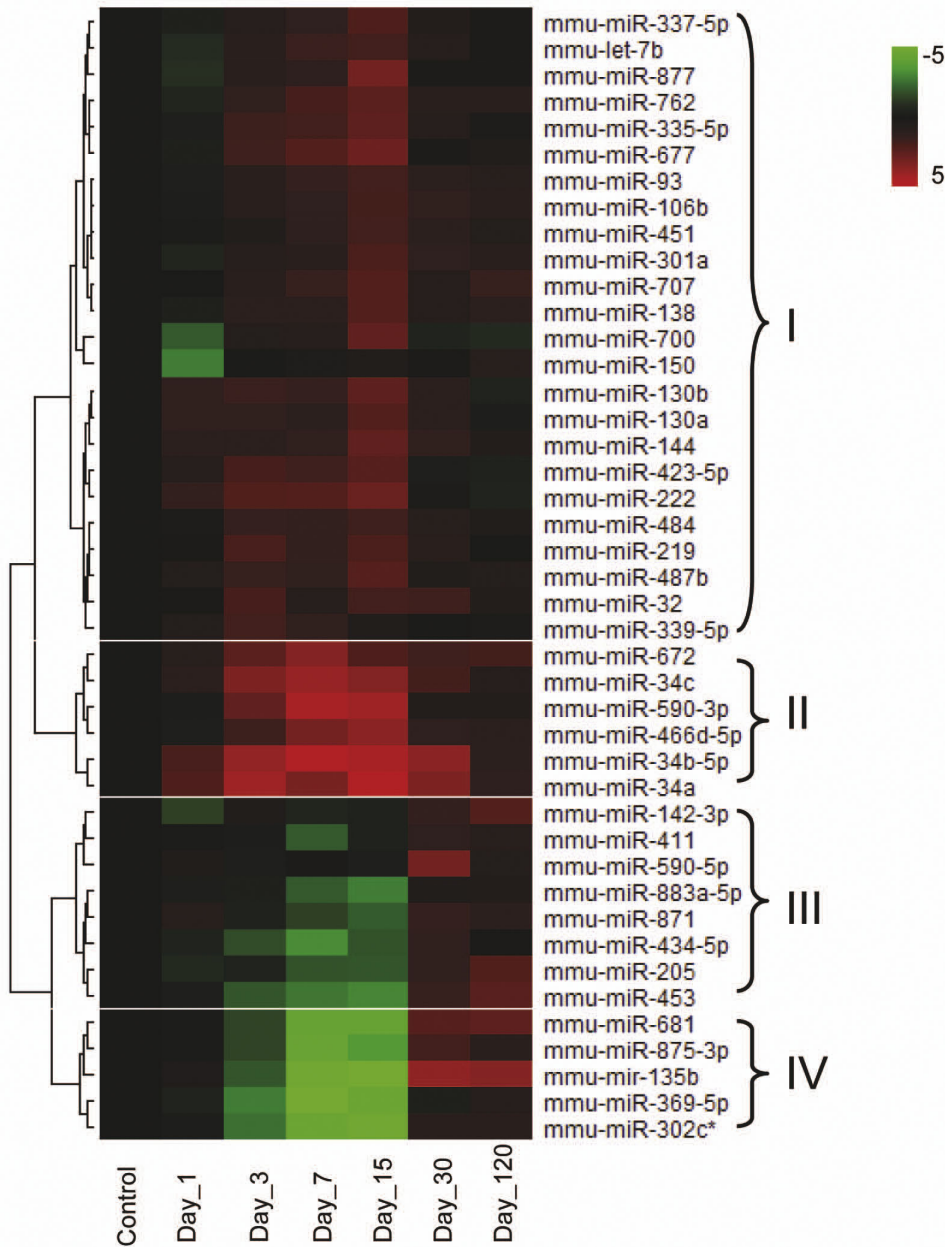
**Hierarchical clustering analysis of 43 deferentially expressed miRNAs**. Fold change for each miRNA at each time point after ENU treatment was determined relative to the vehicle-treated controls and logarithmically transformed (base 2). Hierarchical cluster analysis was conducted using a Euclidean distance-calculating and Ward linking method. Up-regulated miRNAs are shown in red; down-regulated miRNAs in green; and no-changes in black. Four clustering groups were identified and named as I, II, III, and IV, respectively.

**Figure 5:**
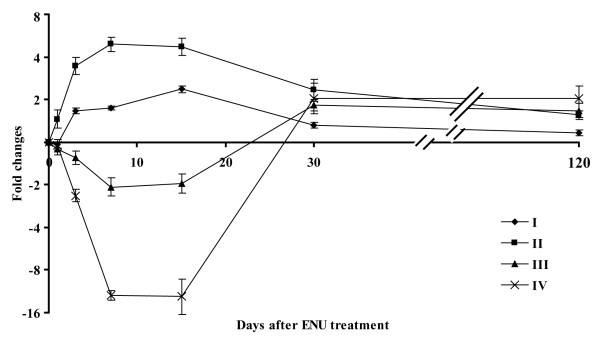
**Average fold changes of the 4 clustering groups with the different sampling times**. The fold changes of all miRNAs in one clustering group were averaged and plotted with its standard errors of the mean against the time points after ENU treatment.

The 43 DEMs were grouped using hierarchical clustering methods based on their logarithmically transformed fold changes. The result is shown in Figure 4. The DEMs were clustered into four groups. Groups I and II included the DEMs whose expression was mainly up-regulated. While the DEMs in Group II were up-regulated across all time points, those in Group I showed slightly down-regulated in one or two time points. Groups III and IV composed of DEMs whose expressions were down-regulated. Day 7 and 15 clearly show the highest fold changes compared to other time points, indicating miRNA expression reached maximal levels or changes between the two time points (Figure 4 and 5).

### In silico Functional analysis of the DEMs

A literature search of the PubMed database was conducted using the gene name of each DEM without considering species difference since miRNA functions are relatively conservative across different species [19]. Twenty seven out of 43 of the DEMs have been functionally investigated by biological experiments (Table 1). Many of these miRNAs were dysregulated in tumors or tissues exposed to carcinogens. They played roles in cell proliferation, cell cycle regulation, cellular transformation, immune response, invasion, apoptosis, tissue development and growth. Some of the DEMs are putative tumor suppressors or oncogenes such as miR-34a (see literature cited in Table 1).

**Figure 6:**
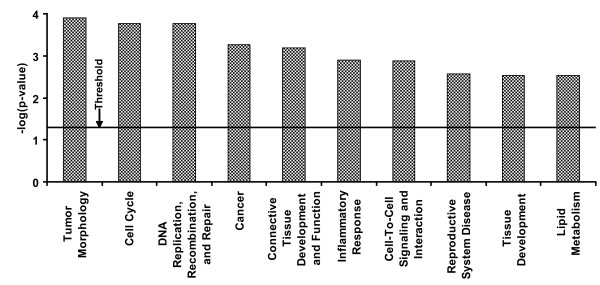
**The top 10 biological functions altered by ENU treatment according to the *p *values**. The target genes of the differentially expressed miRNAs at post-treatment days 7 and 15 were computationally predicted by miRanda algorithm. The top 5% predicted genes were utilized for functional analysis using Ingenuity Pathway Analysis system. The threshold (*p *= 0.05) indicates whether the functions are significantly changed by the treatment or not. If a *p *value of a function is less than the threshold, the function is considered as significantly changed.

Although the functions of the DEMs are highly related to tumorigenesis according to the reports, the studies on the miRNA functions were conducted in different species and various biological systems. To explore the functional relationship between miRNA expression changes and ENU treatment, we used the top computationally-predicted target genes of these DEMs according to the sequence complementarity between miRNA and their target mRNAs for our functional analysis. Such approaches have been widely used for functional analysis of miRNA target genes since miRNAs exert their gene regulatory activity primarily by base pairing to the 3' UTR of their target mRNA, leading to mRNA degradation or translational inhibition [20]. Thirty six DEMs at PTDs 7 and 15 were used for this analysis because the principal component analysis showed that the global miRNA expression was altered by ENU treatment at these two time points. The selected target genes of the DEMs were input into IPA, and the related functions and pathways were determined and are provided in Figure 6 and Additional File 3. The most relevant biological functions putatively controlled by the ENU-induced DEMs are related to mutagenesis and tumorigenesis, involved in tumor/cancer formation, cell cycle and DNA repair, as well as other ENU-toxicity related functions.

## Discussion

To explore the temporal response of miRNA to treatment of genotoxic carcinogens, mice were administrated with one dose of 120 mg/kg ENU, which can significantly induce tumors and mutations in mouse liver [13]. The ENU treatment resulted in temporal changes in miRNA expression in mouse liver and the altered miRNAs are functionally related to the carcinogenicity and mutagenicity of ENU according to the *in silico *functional analysis of the DEMs.

### Temporal changes in miRNA expression after ENU treatment

miRNA aberrant expression has been found to be a common feature of tumor malignancy [21]. Several studies on carcinogen-altered miRNA expression have been conducted and showed that chronic or subchronic carcinogen exposure can dysregulate miRNA expression. Long-term exposure of female Fisher 344 (F344) rats to a tamoxifen-containing diet led to alterations in the miRNA expression profile in liver tissue prior to tumor formation [22]. Sprague-Dawley rats treated with 2-AAF for 12 or 24 weeks exhibited disrupted regulation of the miR-34a-p53 feed-back loop and substantial deregulation of expression of miR-18, miR-21, miR-182, and miR-200 family miRNAs [11]. Male F344 rats continuously fed with NNK for up to 20 weeks resulted in alteration of miRNA expression in the lungs of rats [9]. Although these studies demonstrated that carcinogen exposures were able to alter expression of certain miRNAs, such experiments could not provide information on temporal response of miRNAs to carcinogen exposure. The alteration of miRNA expression by these subchronic to chronic treatments do not provide the response period of miRNAs to treatment. The changes could result from the cumulative effects of persistent long-term chemical exposure or from a short term response. At present, the duration of miRNA response to exposure to a genotoxic carcinogen in animals or humans is unknown. In this study, mice received only a single dose of a potent genotoxic carcinogen ENU and were sacrificed at different time points so that temporal response of miRNA expression to the treatment can be determined. The results clearly demonstrate that exposure of mice to a single dose of ENU can cause distinctive alterations in miRNA expression at the different sampling times after the ENU treatment (Table 1 and Figures 1, 2, 3 and 4).

Sampling time after the ENU exposure appears to be a very important parameter in miRNA expression in both the amount of DEMs and their fold-change levels. Principal component analysis indicates that mouse livers sampled at 7 and 15 days after ENU treatment display distinctive miRNA expression patterns compared to controls and other sampling time points (Figure 1). Three DEMs were found one day after the ENU treatment. The number of DEMs increased with the sampling time, reaching the peak at PTD 15 with 32 DEMs. The amount of DEMs declined after 15 days and only 5 DEMs were identified at PTDs 30 and 120 (Figure 2). The magnitude of the DEMs' expression alteration was also changed with time. Most of the DEMs had their highest fold-changes at PTDs 7 or 15 (Figures 4 and 5). Hierarchical clustering analysis of the DEMs divided them into four groups according to the change direction and intensity of these DEMs' expression alteration. The DEMs in Groups II and IV responded to the ENU exposure quickly, indicating that some miRNAs can be changed by the treatment within a few days. However, regarding the alteration of miRNA expression induced by the ENU treatment, both the amount and the change magnitude of the DEMs peaked at PTD 15.

The pattern of temporal changes in miRNA expression after ENU treatment is different from previous reports on gene expression changed by ENU treatment [17]. In the previous studies, gene expression changes in both amount and intensity peaked at 4 hours and declined at 20 hours, 14 and 28 days after administration of ENU. The mechanism(s) for the difference between the temporal changes induced by ENU in miRNA expression and gene expression is unknown. It is possible that many genes, such as DNA repair genes, can directly respond to the DNA damage caused by ENU exposure whereas miRNAs as posttranscriptional regulators for gene expression may respond indirectly to ENU insults via the alteration of gene expression [23]. Also, turnover rates for mature mRNAs and miRNAs could play a role in the difference. Mature miRNAs generally have lower turnover rates and exist longer than mRNAs [24].

The pattern of the temporal expression change could result from biological responses of liver miRNAs to the genotoxic and cytotoxic effects of ENU. miRNA expression may vary between different biological mechanisms like DNA adduct formation by ENU, cell death induced by the DNA damage, and cell proliferation due to the cell death. The DNA damage induced by ENU is very fast, occurring within hours after treatment [25]. However, the DNA repair and cell proliferation processes could take a few weeks [26]. Some miRNAs like miR-34a could respond to the DNA damage quickly and exhibited dysregulation within one day while most miRNAs that function mainly as regulators for cell differentiation and proliferation altered their expression only after several days.

### In silico pathway analysis of the functions of the ENU-induced DEMs

ENU, as a potent monofunctional ethylating agent, reacts directly with the nucleophilic nitrogen and oxygen atoms in DNA and with the oxygen atoms in the backbone phosphates, forming various ethylated products including *N*^7^-ethylgunine, *N*^3^-ethyladenine, *O*^6^-ethylguanine, *O*^2^- and *O*^4^-ethylthymine, and *O*^2^-ethylcytosine [16,25]. Once formed in the DNA, these products can result in cell death, like apoptosis [27] or become the substrate of cellular repair processes of various kinds, such as specific dealkylation by the *O*^6^- alkylguanine-DNA-alkyltransferase (AGT) [28], removal of *N*^7^-alkylguanine or *N*^3^-alkylguanine by specific glycosylases [29], or removal *O*^4^-alkylthymine by the more general action of the nucleotide excision repair system [30]. Also, ENU is known to induce cell proliferation [31]. If miRNAs are involved in the regulation of the genes that are related to genotoxic functions like cell proliferation, cell cycle arrest, apoptosis, and DNA repair, expression of miRNAs related to these functions should be changed by the ENU treatment. Indeed, our literature search results indicate that most of the DEMs induced by ENU treatment function as regulators for cell cycle arrest [32], cell proliferation [33], apoptosis [34], DNA repair [35] and other biological processes related to ENU cytotoxicity, genotoxicity and carcinogenicity (Table 1).

Because each miRNA can regulate many target genes and several miRNAs may affect a single gene, it is important to analyze the functions of all DEMs together. Computational approaches have been a major focus in determining the general principles that are thought to govern miRNA target recognition and mode of action. In this study, the target genes computationally predicted by the miRanda algorithm were used for functional analysis. The miRanda algorithm was developed by the Sanger Institute and is widely used in miRNA studies [36]. The top 5% target genes of all of the DEMs were selected and used for IPA functional analysis. The top functions affected by the ENU exposure are tumor morphology, cell cycle, DNA replication, recombination and repair, and cancer. These biological functions identified by the analysis show that the DEMs are related to ENU-induced carcinogenesis (Figure 6). For example, many genes involved in DNA repair or response to DNA damage can be dysregulated by ENU treatment [37] and the miRNAs that target these genes could change their expression to regulate these functional processes.

### MiR-34 family might have the potential to be explored as a biomarker for genotoxin exposure

Our results indicate that some miRNAs responded to ENU treatment with a wide temporal range. These miRNAs might have the potential to be used as biomarkers for predicting the genotoxic carcinogenicity of chemicals. Among these miRNAs, the miR-34 family is worth special attention. All of the 3 miRNAs were significantly changed at four different time points (Figure 3). Their expressions were enhanced by 3.21-fold (miR-34a), 3.11-fold (miR-34b) and 2.37-fold (miR-34c) on PTD 1 and the fold changes continued to increase and peaked at PTDs 7 or 15. The miR34 family genes are the direct transcription targets of tumor suppressor *p*53 [32,38]. miR-34b and miR-34c are encoded by the same primary transcript from chromosome 11 in human or chromosome 9 in mouse while miR-34a is located in a different chromosome [35]. The promoter region of miR-34a and miR-34b/c each contain a palindromic sequence that matches the canonical *p*53 binding site and can be bound by *p*53 as shown by chromatin immunoprecipitation [32]. Interestingly, our results found that miR-34b and miR-34c changed in correlated manner at all the sampling time points (Figure 3). miRNAs in miR-34 family play important roles in various *p*53-initiated biological processes. Up-regulation of miR-34a and miR-34b/c caused a cell-cycle arrest in the G1 phase [32]. miR-34b/c inhibits cell proliferation and colony formation in soft agar [39]. Introduction of miR-34a and miR-34b/c into primary human diploid fibroblasts induces cellular senescence [35]. Re-expression of miR-34a in tumor cells induced apoptosis [38]. These biological processes controlled by miRNAs in the miR-34 family are related to ENU cytotoxicity, genotoxicity, and carcinogenicity. Our results indicate that the miR-34 family of miRNAs seems to have the potential to be valuable biomarkers for toxicological application.

## Conclusions

Our study indicates that one dose treatment of ENU, a chemical inducing tumors and mutations, resulted in deregulation of a large number of miRNAs. In silico functional analysis suggested that these miRNAs were related to ENU mutagenesis and carcinogenesis in the mouse liver. The deregulation of ENU-induced miRNA expression changed with time and peaked at day 15 after the treatment. The findings suggest that one to two weeks after ENU exposure is the best time for miRNA expression sampling. Moreover, miRNAs in the miR-34 family worth further study to explore their potential as biomarkers for exposure of genotoxic carcinogens.

## Methods

### Animal treatment

The animal treatment protocol and mutant frequency analysis was described previously [13]. Briefly, six-month-old female Big Blue mice were injected intraperitoneally with a single dose of 120 mg/kg body weight ENU (CAS# 759-73-9, Sigma, St. Louis, MO) or the vehicle dimethylsulfoxide (DMSO, Sigma) in 1 ml/kg body weight (0.1%). For ENU treatment, groups of 4 or 5 animals were sacrificed on PTDs 1, 3, 7, 15, 30, and 120. For the vehicle treatment, 4 and 3 animals were sacrificed on PTDs 1 and 30, respectively. DMSO is not carcinogenic and mutagenic [40]. It has showed no effects on gene expression in the dose that we used [41]. Also, our previous study demonstrated that DMSO did not change mutant frequency in mouse liver at different sampling times [13]. Therefore, the 7 control samples were grouped together as a common control group for the treatment groups sampling at the different time points to increase the statistical power. The tissues were isolated and frozen at -80°C. The liver samples were used for this study. All animal experiments were conducted by following the recommendations set forth by our Institutional Animal Care and Use Committee.

### miRNA isolation

About 60 mg of liver tissue was cut from each frozen liver sample and suspended in RNA*later*-ICE (Ambion Inc., Austin, TX). The tissue pieces were transferred to 600 μl RNA lysis/binding buffer and minced using Tissue Tearor™ (BioSpec Products Inc., Bartlesville, OK). miRNAs were isolated using mirVana™ miRNA isolation kit (Ambion) that specifically captures small RNAs with length of less than 200 nucleotides. The isolated RNAs were resolved in 100 μl nuclease-free water (Ambion). RNA concentrations were determined using NanoDrop 1000 Spectrophotometer (NanoDrop Technologies, Wilmington, Delaware). The quality of RNA samples was characterized on an Agilent BioAnalyzer (Agilent Technologies, Santa Clara, CA) using an RNA6000 Nano Chip (Agilent).

### PCR Array analysis of miRNA expression

Two hundred nanograms of enriched small RNA were converted into cDNA using RT^2 ^miRNA First Strand Kit (SABiosciences Corporation, Frederick, MD). The cDNAs were mixed with 2 × RT^2 ^SYBR Green PCR Master Mix (SABiosciences) and dispersed into 384-well Mouse Genome miRNA PCR Array (MAM-3100E, SABiosciences) with 10 μl/well reaction volume. The PCR array contained a panel of primer sets for 376 mouse miRNAs, four small RNAs as the internal controls and four quality controls. The real-time qRT-PCR was performed on a 7900 real-time PCR system (Applied Biosystems Inc., Foster, CA) with following cycling parameters: 95°C for 10 mins, then 40 cycles of 95°C for 15 s, 60°C for 30 s and 72°C for 30 s. SYBR Green fluorescence was recorded from every well during the annealing step of each cycle. The threshold cycle (Ct) value of each sample was calculated with software SDS 2.3 (Applied Biosystems). To calculate Cts, we set the threshold line as 0.15 and kept it the same across all of the analyses. The baseline was automatically defined by the software.

### Normalization and statistical analysis

Normalization and statistical analysis of miRNA expression were conducted using SABiosciences Online PCR Array Data Analysis Web Portal. MiRNA expressions were compared between the treatment group at each time point and the control group. The ΔΔCt method was utilized to calculate the fold change (FC). Four genes, snoRNA251, snoRNA202, snoRNA142, and U6 in the PCR arrays, were averaged as the endogenous control and the vehicle control group was used as external control to normalize each sample. The formula: FC = 2^ [-(mean of ΔCt values of treated samples - mean of ΔCt values of control samples)] was used for up-regulated gene, while FC = - 2^ (mean of ΔCt values of treated samples - mean of ΔCt values of control samples) was used for the down-regulations. T-tests were used to calculate the *p *value to determine whether there is a significant difference for miRNA expression between the control and the treatment groups for each miRNA at each time point. miRNAs with *p *< 0.01 and the absolute value of FC >2.0 were considered as DEMs.

### Principal component analysis and hierarchical clustering analysis

Principal component analysis of expression profiles of all miRNAs from each time point after the ENU treatment was conducted using the autoscaled method within ArrayTrack [42]. The normalized ΔCt values were used for this analysis and the analysis was performed without filtering any miRNAs.

To examine types of expression changes, hierarchical clustering analysis was performed using R software http://www.r-project.org/. The miRNAs whose expressions were significantly differentially expressed at least at one sampling time were used for the clustering. The FCs for control samples were set to zero. Euclidean and Wards methods were used for distance-calculation and linkage, respectively.

### In silico Functional analysis of the DEMs

The miRanda database was used for identification of DEMs' target genes http://microrna.sanger.ac.uk/sequences/. The top 5% of the most reliable predicted target genes of the DEMs at PTDs 7 and 15 were selected according to the *p *values given in the database. A total of 1376 genes were determined as the predictive target genes of these DEMs. The selected target genes were then input into Ingenuity Pathway Analysis (IPA) software (Ingenuity Systems, Inc., Redwood City, CA). Ingenuity Core Analysis and Knowledge Base was used as the reference set. IPA interpreted the genes in the context of biological processes, pathways and molecular networks and defined the most relevant biological functions of the predicted genes of the DEMs.

### TaqMan qPCR confirmation of the temporal expression changes of miR-34 family miRNAs

TaqMan MicroRNA Assays were used to confirm the temporal expression changes of 3 miR-34 family members, mmu-miR-34a, mmu-miR-34b-5p, and mmu-miR-34c, as well as a miR-762 family member, mmu-miR-762. The experiment also severed as the verification of the RT^2^-mouse miRNA PCR array assay. The TaqMan miRNA assay kits were purchased from Applied Biosystems (Foster City, CA). The same small RNA samples used for the PCR arrays were also used for the TaqMan miRNA assays. The experiment was performed by following the manufacturer's protocol. In brief, each 10 μl reverse transcription (RT) reaction contained 44 ng of small RNA, 50 nM stem-loop RT primer, 1 × RT buffer, 0.25 mM each of dNTPs, 3.33 U/μl MultiScribe™ reverse transcriptase and 0.25 U/μl RNase inhibitor. The RT reactions were incubated in a GeneAmp PCR System 9700 (Applied Biosystems) for 30 min at 16°C, 30 min at 42°C, followed by 5 min at 85°C, and then held at 4°C. Each real-time PCR reaction (10 μl volume) containing 0.78 μl of RT product, 5 μl of 2× TaqMan Universal PCR Master Mix, and 0.5 μl TaqMan MicroRNA assay (the mixture of TaqMan probe, forward primer, and reverse primer). The PCR reaction was conducted in an Applied Biosystems 7500 Fast Real-Time PCR System at 95°C for 10 min, followed by 40 cycles of 95°C for 15 sec and 60°C for 1 min. The threshold cycle (Ct) is defined as the fractional cycle number at which the fluorescence exceeds the fixed threshold of 0.02. Four samples at each time point were used for the TaqMan confirmation.

### Accession numbers

All PCR array Ct raw data are available through Gene Expression Omnibus (Series accession numbers: GSE20248).

## Authors' contributions

ZL carried out the experiments and wrote the manuscript. WSB and SLD participated in the PCR array experiments. YW provided critical assistances to improve PCR array performance and reproducibility. LG provided help for performing real-time PCR array. LS participated in data analysis. TC originally designed this study and wrote the paper. All authors have read and approved the final manuscript.

## Supplementary Material

Additional file 1 The normalized Ct values (dCt) for the control samples and treatment samples at different days after ENU treatment. Click here for file

Additional file 2 The p values and fold changes of microRNAs at different days after ENU treatment. Click here for file

Additional file 3 The biological functions related to the top 5% targets of DEMs. Click here for file
